# Clinical risk management in mental health: a qualitative study of main risks and related organizational management practices

**DOI:** 10.1186/1472-6963-13-44

**Published:** 2013-02-04

**Authors:** Matthias Briner, Tanja Manser

**Affiliations:** 1 ETH Zurich, Centre for Organizational and Occupational Sciences, Weinbergstrasse 56/58, 8092, Zurich, Switzerland; 2 Lucerne School of Business, Lucerne University of Applied Sciences and Arts, Zentralstrasse 9, 6002, Lucerne, Switzerland; 3 Department of Psychology, University of Fribourg, Rue P.-A. de Faucigny 2, 1700, Fribourg, Switzerland

**Keywords:** Patient safety, Clinical risk management, Organizational risk management, Mental health care, Psychiatry, Qualitative analysis

## Abstract

**Background:**

A scientific understanding of clinical risk management (CRM) in mental health care is essential for building safer health systems and for improving patient safety. While evidence on patient safety and CRM in physical health care has increased, there is limited research on these issues in mental health care. This qualitative study provides an overview of the most important clinical risks in mental health and related organizational management practices.

**Methods:**

We conducted in-depth expert interviews with professionals responsible for CRM in psychiatric hospitals. Interviews were transcribed and analyzed applying qualitative content analysis to thematically sort the identified risks.

**Results:**

The main concerns for CRM in mental health are a) violence and self-destructive behavior (i.e. protecting patients and staff from other patients, and patients from themselves), b) treatment errors, especially in the process of therapy, and c) risks associated with mental illnesses (e.g. psychosis or depression). This study identified critical differences to CRM in hospitals for physical disorder and challenges specific to CRM in mental health. Firstly, many psychiatric patients do not believe that they are ill and are therefore in hospital against their will. Secondly, staff safety is a much more prominent theme for CRM in mental health care as it is directly related to the specifics of mental illnesses.

**Conclusions:**

The current study contributes to the understanding of patient safety and raises awareness for CRM in mental health. The mental health specific overview of central risks and related organizational management practices offers a valuable basis for CRM development in mental health and an addition to CRM in general.

## Background

Understanding and improving patient safety is a growing concern, particularly following the publication of the Institute of Medicine reports “To err is human” [1], “Crossing the quality chasm” [2] and the NHS’s “Organisation with a memory” [3]. These reports highlight that between 3.7-16.6% of patients admitted to hospitals suffer an adverse event, at least half of which are preventable. Such adverse events can result in unnecessary injury or death as well as enormous economic costs. Despite being ostensibly concerned with patient safety and minimizing risks in health care, a systematic approach to patient safety or a systematic organizational management of clinical risks is difficult to implement and therefore, seldom seen [4-6].

Nevertheless, research and knowledge on patient safety, have increased rapidly and improved many aspects in acute medical health care settings [7,8]. However, in mental health care, there is a “lack of awareness of the issues as well as a shortage of research and information on the topic” [9], p. 39]. A comprehensive literature review highlights an inconsistency in basic patient safety concepts in mental health (e.g. defining and calculating adverse events), as well as a scarcity of high quality patient safety research in mental health [10]. Due to the resulting lack of patient safety principles specific to mental health care, concepts and strategies from acute medical health care settings are frequently adopted. This may be appropriate for some aspects, but mental health care differs from medical patient care in patient population and illnesses, as well as in historical and institutional contexts. There are also unique patient safety issues in mental health care that require further consideration [cf. [10-12], especially with regard to clinical risks. While medication related risks, such as medication mix-up or delivery of wrong dose, are found in acute medical care and mental health [e.g. [13], specific risks, such as suicide, violence and self-harm prevail in mental health [14]. To date, an overview of the spectrum of clinical risks found in mental health and the organizational risk management practices currently applied is lacking. Publications mostly discuss specific risks, such as violence, and do not offer an integrated view e.g. (for suicidal or violent patients, see [15,16]). Also, the traditional focus of the management of clinical risks in mental health care was located at the individual instead of the organizational level and was therefore narrowly “considered the business of predicting and preventing dangerousness” of patients [14], p. 3].

Furthermore, a systematic clinical risk management (CRM) can play a crucial role in enabling health care organizations to assess, manage, and contain risks related to patient safety and aims at reducing or eliminating harm to patients [8,17]. The more complex an organization, the greater the need for CRM. This is especially true for psychiatric hospitals, where the challenges to patient safety are varied and the connection between patient and staff safety is closer than in hospitals for medical complaints [e.g. [18,19].

To gain a systematic and comprehensive understanding of CRM in mental health, this study aims to provide an overview of clinical risks and related management practices in mental health. This is an important step in deepening our knowledge of patient safety and in supporting psychiatric hospitals to optimize their clinical risk management and to ultimately improve the health care system, for the mentally ill [13].

## Methods

### Sample, setting, and data collection

This study used semi-structured expert interviews to identify clinical risks in mental health care and organizational risk management practices. Expert interviews are a very useful instrument for innovative research taking into account the expert status of the interviewee; they allow for collecting the interviewees subjective experiences and interpretations regarding a predefined specialized topic [20]. The semi-structured form supports comparability between the interviews, yet allows for the inclusion of not anticipated, but important issues [21]. Interviewing persons with patient safety expertise in mental health care, therefore, is a valuable source of in-depth information that is urgently needed to expand research in this field where currently there is little research available [9].

The interviewees were selected following a national study on CRM in Switzerland in 2007/08 [see [4,17]. The sampling technique was purposive: all 11 experts were responsible for the coordination of CRM in their psychiatric hospital and had considerable knowledge and experience in the field of patient safety in mental health care. Eight of these experts had worked for more than five years in their respective institutions; six hospitals were public, five were private. Four hospitals had fewer than 100 beds (all private hospitals), two had 100–200 beds (all public), and five had over 200 beds (four public, one private). Participation was voluntary and did not affect respondents physically or mentally. All responses were de-identified. The research did not include any patients and is in line with the WMA Declaration of Helsinki - Ethical Principles for Medical Research Involving Human Subjects. Such research does not require ethics approval in Switzerland, as mere surveys in the sense of opinion surveys or interviews are not counted as research on humans (see http://www.vpf.ethz.ch/about/commissions/EK).

Interviews were carried out by an experienced researcher (in most cases accompanied by an assistant) between June and September 2008 in the interviewees’ offices in the respective psychiatric hospital. In three interviews, additional personnel participated (nursing resp. medical head, responsible person for work safety). Interviews lasted between 80 and 160 minutes and were audio recorded.

The interview manual was developed as part of the project, “Clinical risk management in Swiss hospitals” [17]. It was based upon results from a literature review on CRM and was critically examined by an expert panel consisting of 11 patient safety experts (comprised of the persons in charge of patient safety and/or quality of five main Swiss healthcare institutions, the president of the Swiss Society for Quality Management in Health Care, the head of quality of a major reinsurance company, and four clinical experts with a proven record of accomplishment in patient safety. For details see [17]). The manual included exploratory questions on tasks, content and organization of CRM (e.g. “What is the meaning of CRM and patient safety in a psychiatric hospital?”), and questions on future developments pertaining to CRM (e.g. “What activities are planned in the next 12 months in the area of CRM/patient safety in your psychiatric hospital?”). It also comprised a structured review of the results of a 2007/2008 survey of CRM that is not part of the current study. The results from the survey are published in Briner, Manser and Kessler [4].

### Data analysis

Interviews were transcribed verbatim and in their entirety, which is crucial for an explorative study, as protocols from memory or summaries reduce information in a methodologically uncontrolled way [22]. To achieve uniformity, the same researcher transcribed all the interviews. The transcripts were analyzed applying qualitative content analysis [23]. This method qualifies for semi-structured expert interviews as it is used for coding text with a predefined coding system which can be refined and completed with new themes emerging in the interviews [22,23]. Our initial coding system used categories which were defined following the literature review of CRM. It allows for organizing, sorting and retrieving the coded text passages. This technique for guiding the analysis of qualitative data relying on prior research had proven valuable in previous studies, for example, identifying and categorizing errors in mental health [13].

The coding was performed using the program MAXQDA2010 that was developed particularly for computer-assisted analysis of qualitative data. To begin the qualitative content analysis, two primary coders (MB and an assistant) coded the transcripts. The specific risks and related organizational risk management practices were assigned to the appropriate categories. Meaningful units (whole or part sentences) were defined as units of analysis. Results were compared between coders to deepen the understanding of the categories and to achieve consensus. The primary coders then reviewed all interviews a second time to refine, expand, bridge or eliminate categories for the purpose of fully describing risks and their organizational management. Inter-rater agreement was calculated to measure the extent different coders agreed upon which text passages were assigned to which categories [23]. Therefore, the spontaneously mentioned risks (risks that, at the beginning of each interview, were spontaneously mentioned to the question, “What is the meaning of CRM in a psychiatric hospital?”, were assigned to the respective categories by the three coders (MB and two assistants) independently. These spontaneously mentioned risks offer a heuristic [fast and frugal judgment, cf. [24] of frequent or obvious risks in mental health. Overall, an inter-rater agreement of 81% was reached. The remaining disagreements were discussed between the three coders until a consensus was reached. Where there was ambiguity, the coding system was adapted and refined accordingly. The two primary coders coded all interviews a third time using this refined coding system in order to reach a final assignment of text passages to categories. The results were further processed independently from the original text and codes were summarized thematically. The frequencies of risks mentioned across all interviews, as well as the spontaneously mentioned risks, were counted to indicate the relative importance of individual risk categories (see results and Table 1). Similar methods were also used by Brickell and McLean [9] for their qualitative analysis of expert perspectives on patient safety in mental health. As management of specific risks was often mentioned at the same time as the risk, it was coded simultaneously.

**Table 1:** Detailed overview of the main risk themes of clinical risk management in mental health care

**Risks**	**Main- / subcategories**	**Risk description**	**Number spont.**	**Number total**	**Total spont.**	**Total overall**	**Mentioned organizational CRM practices (selection)**
A	Clinical risks	General statements about clinical risks without the mention of a specific risk	1 of 11	1 of 11	1	2	
A1	Clinical risks specific to mental health care	Clinical risks specific to mental health care, i.e. risks that occur only (or predominantly), or are typical, in mental health care	1 of 11	3 of 11	1	5	· Admission interview generally considered important
A1.1*	*Violence / aggression*	General statements about risk themes regarding violence or aggression (physical/psychological). Specific risks are listed in the sub-categories	8 of 11	*10 of 11*	12	*42*	· Aggression management training
							· Violence risk assessment (e.g. Brøset -Checklist)
							· Compulsory measures, sensory deprivation, seclusion
							· Structural preventive measures
							· When too dangerous: prison and external supervision
A1.1.1	*Self-destructive behavior*	Self-destructive behavior of a patient (e.g. suicide, suicide attempts, self-injury and self-harm: cutting.)	9 of 11	*11 of 11*	11	*51*	· Good anamnesis, pre-admission interview
							· Clarify during admission interview and other consultations
							· Intensive support/monitoring
							· No-suicide contract
							· Closing of the ward
							· Good follow-up care and debriefing
A1.1.2*	*Compulsory measures*	Seclusion, restraint, etc. when mentioned as a risk or as a measure against a risk	4 of 11	*9 of 11*	4	*31*	· Training
							· Standardized procedures
							· Inform beforehand
							· Observation and/or seclusion room
							· Debriefing
A1.1.3*	Next of kin, risks from the outside	Assault/threats from next of kin or from outside	1 of 11	2 of 11	1	4	
A1.1.4*	Violence with or towards objects	Any form of violence with objects (e.g. weapons, lighters); also violence towards objects (e.g. to destroy furniture)	0	2 of 11	0	5	· No dangerous objects and infrastructure
							· Nonflammable material in the rooms
A1.1.5*	Physical vs. verbal abuse	General statements specific to verbal abuse (threats) or physical abuse	0	2 of 11	0	3	
A1.2	*Treatment errors*	Treatment errors / treatment risks during treatment procedure, psychotherapy	4 of 11	*11 of 11*	6	*33*	· Standard procedures for consultations
							· Interdisciplinarity
							· Avoid one-to-one consultations
							· Anamnesis with pro-active risk assessment
							· Sufficient staff
							· An ombudsman service that a patient can turn to
A1.2.1	Assaults by staff on patients during the therapeutic process	Assault by a staff member on a patient, especially during the therapeutic setting, that also include, for example, consensual sexual contacts or abuse of power by the therapist	2 of 11	3 of 11	2	6	· Special training
							· Inform patients specifically about this issue
							· Intervision (peer consulting) and supervision
							see also A1.2
A1.2.2	Diagnostic errors	Establishing a diagnosis of a mental illness instead of an underlying physical illness or the misdiagnosis of psychiatric illness, which could result in incorrect treatment	1 of 11	2 of 11	2	3	· Differential diagnosis
							· Additional tools to evaluate physical risks.
A1.2.3	Specific medication risks occurring mainly in psychiatry	All risks related to medication that are (mainly) psychiatric specific, especially: 1) side effects of medication. An important reason why patients do not take their medication. Risk of non-compliance. 2) accumulation, hoarding of medication (e.g. for suicide, substance abuse)	1 of 11	4 of 11	1	7	· Clarify patient’s needs
							· Information about effects and side-effects
							· Information on exercising and nutrition
							· Monitor medication intake
A1.3	*Risks associated with mental illnesses*	Statements about individual illnesses (e.g. addiction, schizophrenia, acute psychosis, mania, depression, anxiety attacks, personality disorder…), that could increase certain risks	4 of 11	*10 of 11*	6	*21*	· Assessment tools
							· Evaluate contractual capacity
							· Intensive support
A1.3.1	*Hospitalization against the will of the patient*	Hospitalization against the will of the patient and/or against the will of next-of-kin. Also lack of insight regarding illness	3 of 11	*8 of 11*	3	12	· Non-voluntary hospitalization, compulsory measures
							· Admit voluntary patients only
							· Involuntary commitment
A1.3.2	Substance abuse	Drugs, smuggling of substances	1 of 11	4 of 11	1	4	· Search patients
							· Sign addiction contract
A1.4	*Absconding*	Patient escapes from psychiatric clinic. This can happen for various reasons, e.g. hears imperative voices, suicidal tendency	3 of 11	*6 of 11*	4	9	· Internal transfer of patient
							· Closing of ward
							· Search by police
A2	Common clinical risks	Common clinical risks occurring in mental health care, but that are not specific, e.g. medication errors, infections. There are also grey areas such as with falls					
A2.1	*Medication risks*	Common medication risks not specific to mental health care, e.g. confusing medication.	5 of 11	*9 of 11*	7	*33*	
A2.2*	*Infections and hygiene*	Infections, disease transmission.	5 of 11	*7 of 11*	5	*26*	· Hygiene, hygiene standards, everything that protects against infection
A2.3	Falls	Falls and their consequences. Likely to be very important with withdrawal symptoms and in geronto-psychiatry	1 of 11	5 of 11	1	12	
A2.4*	*Staff risks*	Lack of staff, high workload. Staff absenteeism due to illness (maybe especially high in mental health care?) Shift change, etc. → a latent condition that can increase risk of errors	1 of 11	*9 of 11*	2	*28*	· Absence management, reintegration, training
							· Hire sufficient staff
							· Attractive training programs
A2.5	Technology and equipment	Technical equipment used in the treatment of patients	2 of 11	3 of 11	2	4	· Control procedures and repair of electronic equipment
							· Correct application and periodic maintenance
A2.6	High rate of internal patient transfers	Patient transfers that represent risks at the interface (change of primary caregiver, organization of transfer, etc.)	0	2 of 11	0	3	
B*	*Other risks (non-clinical)*	Common, non-clinical risks (e.g. financial, structural risks, risks relating to image, etc.) e.g. fire, data protection, that represent only an indirect clinical risk	6 of 11	*11 of 11*	14	*47*	
C*	*Risks for the staff (Staff safety)*	Explicit risks that mainly concern staff members	1 of 11	*11 of 11*	2	*38*	· Preventive measures (e.g. raising awareness, staff training)
							· Active measures (e.g. de-escalation techniques, compulsory measures)
							· Follow-up measures (e.g. debriefing, care teams)

Description of the individual columns in Table 1:

· *Risks*: numbering of risk categories and sub categories (A > A1 > A1.1 etc.).

· *Main category / sub category*: names of the risk categories.

· *Risk description*: explanation of the meaning of the mentioned risk.

· *Number of spontaneously mentioned risks*: shows in how many of the 11 interviews the corresponding risk was spontaneously mentioned at the beginning of the interview.

· *Total number*: shows in how many of the 11 interviews the corresponding risk was mentioned during the interview.

· *Total number of spontaneously mentioned risks*: shows how often the corresponding risk was spontaneously mentioned in total at the beginning of all 11 interviews (multiple mentions in the same interview are included).

· *Overall total of mentioned risks*: shows how often the corresponding risk was mentioned in total during all 11 interviews (multiple mentions in the same interview are included).

· *Mentioned CRM practices (selection):* selection of possible measures on how to deal with the corresponding risk mentioned during the interviews.

The most important risks mentioned in more than half of the interviews or more than 20 times in total are *italicized*.

* Marked with an asterisk are those risks that are important to patient as well as to staff safety.

### Focus group for reflecting interview results

Focus groups offer the possibility to deepen the understanding of results from qualitative studies [cf. [25]. Experts appraise, discuss and reflect upon the findings and thereby add content validity to a study [for the importance of content validity, see [26]. In our case, a focus group took place in August 2011. This comprised four renowned Swiss patient safety experts in mental health care. Each focus group participant was briefed on the study in advance and received a thematically organized tabular overview of the spontaneously mentioned risks found in all interviews (integrated in Table 1, details see above) to prepare for the two-hour focus group session. Three experts were able to participate in the focus group (one was ill and gave written feedback). The three interview coders guided the discussion on the overview of risks in mental health. The discussion was recorded in writing and used to refine the overview of the main risk themes of CRM in mental health (Table 1).

## Results

Our results highlight specifics of CRM in mental health care and give an overview of risks in mental health. The most important organizational CRM practices are presented in conjunction with the corresponding risks, since the experts frequently mentioned them at the same time as the risk. Quotes were translated verbatim into English. The index number (e.g. I1, P3) indicates the interview and the paragraph where the quote was taken from.

### Specifics of CRM in mental health care

It was highlighted throughout the interviews that CRM in mental health differs from CRM in medical health care in important aspects. A major difference lies in the characteristics of psychiatric patients, whose mental illnesses, such as psychosis or depression, entail specific clinical risks. Repeat admission patients are significant as they are characteristic to some kind of diagnoses. In addition, some patients do not believe that they are ill and therefore refuse treatment, whereas patients with an obvious physical injury, such as a broken leg, would not behave in that way. On the other hand, high-risk treatments such as surgery are not found in psychiatry. Therefore, clinical risks such as iatrogenic infections play a somewhat minor role. Overall, CRM in mental health was judged to be less advanced than in medical health care, but a rising awareness of the topic was noted. CRM was seen to support patient safety, but also to be important for staff and family safety: “Service provider and receiver should not be harmed. […] A patient should always leave the ward healthier than on admission” (I3, P17).

### Overview of risks in mental health care

**Figure 1:**
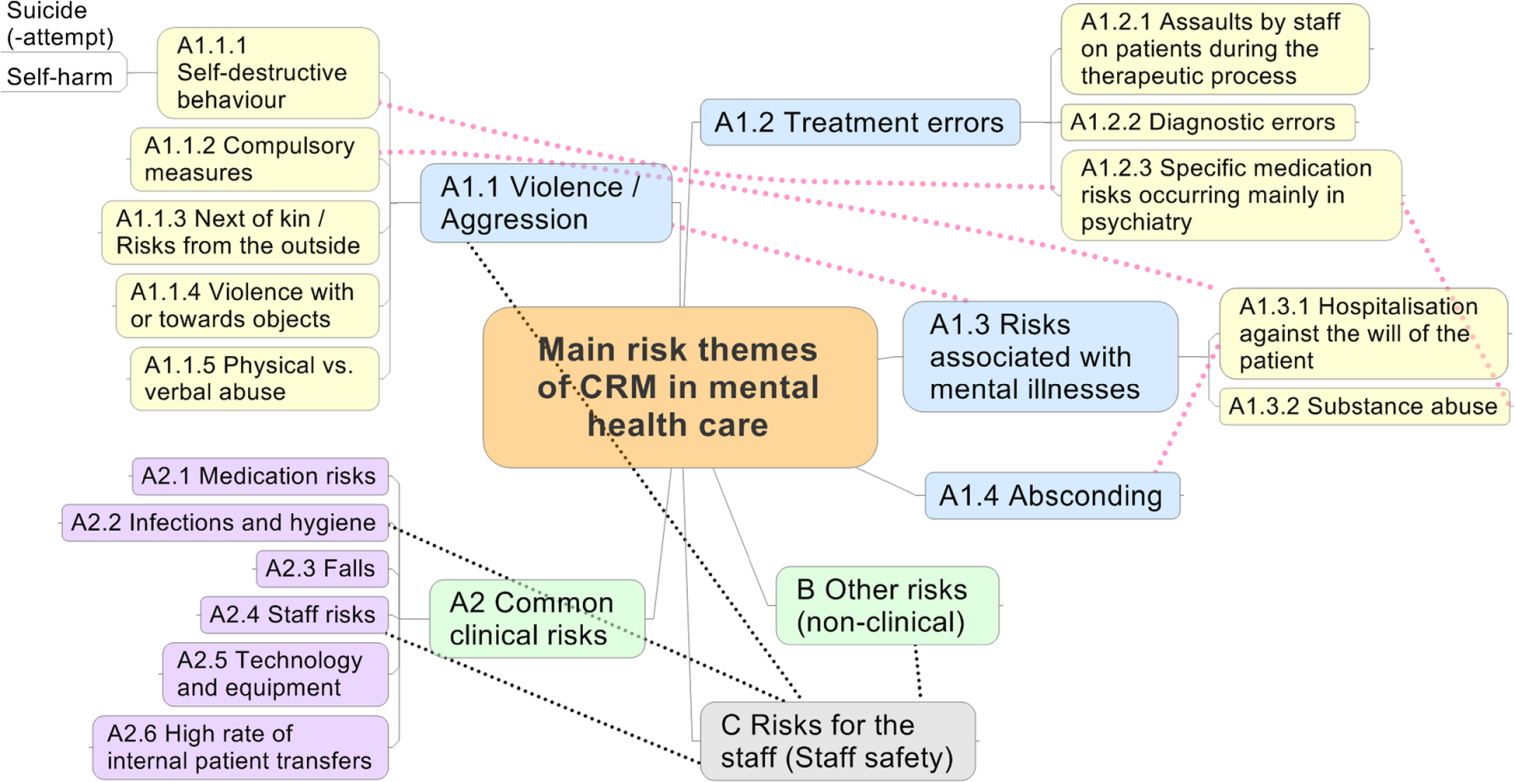
Overview of the main risk themes in mental health care.

Figure 1 provides an overview of the most important risks in mental health. Blue (main categories) and yellow (sub categories) fields show risks that are specific to mental health. Dotted red lines show relations between different categories and dotted black lines show risks that affect staff safety, as well as patient safety. The full overview of the main risk themes of CRM in mental health care mentioned in the interviews and related organizational management practices is given in Table 1.

The focus of this paper is clinical risks specific to mental health care (see A1.1-A1.4 in Figure 1). These were the clinical risks mentioned most frequently in the interviews (n=237), emphasizing their importance. All interviewees also mentioned clinical risks known from medical care that also appear in mental health care (A2, n=106). Additionally, all interviewees mentioned non-clinical risks that are mostly not specific for mental health care (B, n=47). All interviewees also referred explicitly to staff safety (C, n=38), highlighting the importance of this topic in mental health care.

#### Results from the focus group

The participants of the focus group for reflecting interview results agreed that a comprehensive and systematic overview of clinical risks in mental health care is lacking, and that a categorization of these risks is complex and challenging. Aggression and self-destructive behavior were approved as main themes in patient safety in mental health (A1.1). An alternate categorization of risks originating in the patient (peril to self or to others) and risks originating from treatment was outlined, but it was judged not to simplify the categorization.

### A1) Clinical risks specific to mental health care

Violence and aggression (A1.1), treatment errors (especially errors in the process of therapy, A1.2), and risks associated with mental illnesses (A1.3) were the most important clinical risk themes specific to mental health care. An additional theme was leaving hospital against medical advice or absconding from the hospital (A1.4). A thorough admission interview was generally considered as an important measure for managing these risks. Other more specific measures are listed below in conjunction with the corresponding risks.

#### A1.1) Violence and aggression

The greatest focus was on violence/aggression (A1.1, n=141). This is in line with Flewett [14], who describes suicide, violence and self-harm as the most common risks. Violence against others was mentioned 42 times. This means physical (e.g. assault, breach) or verbal/psychological (e.g. threat) violence against fellow patients, staff or other persons (e.g. family members, next of kin). Training and education (aggression management training, fixation technics etc.) were recommended as possible measures against general violence as were violence risk assessments [e.g. prediction instruments such as the Brøset-Violence-Checklist, cf. [27].

Self-destructive behavior (A1.1.1) was mentioned most frequently (n=51), and was also the most frequent spontaneously stated risk. This category comprises suicide, attempted suicide and self-harming (e.g. cutting). All interview partners emphasized the importance of self-destructive behavior: one stated, “I have never seen a patient who could completely exclude suicide” (I5, P20). An assessment of suicidal tendency during admission and in subsequent interviews, no-suicide contracts and good anamnesis as well as architectural protection and intensive support and monitoring of endangered patients, were recommended as possible measures against self-destructive behavior. If something did happen, good follow-up care and debriefing for fellow patients, staff and next of kin is important. Therefore, many psychiatric hospitals developed standard procedures (e.g. procedures after (attempted) suicide).

Compulsory measures (A1.1.2) that are intended to be an activity to calm down violent patients were also seen as a risk (n=31). Compulsory measures are risky as they are usually applied against the will of the patient and sometimes require force to be administered. Training and education, and the use of standardized procedures, were recommended as CRM measures.

Other risks mentioned were violence from the outside (A1.1.3>, e.g. family of patients that threaten other patients or staff), violence with objects (A1.1.4, e.g. weapons) or towards objects (e.g. to destroy furniture etc.) and physical or verbal abuse (A1.1.5, e.g. death threat).

In sum, violence/aggression is linked closely to particular mental illnesses that increase the possibility for violent behavior. This topic is discussed more deeply in the section on risks associated with mental illnesses (see below, A1.3).

#### A1.2) Treatment errors (especially errors in the process of therapy)

The second focus regarding specific clinical risks in mental health care was on treatment errors, especially errors in the process of therapy (A1.2, n=49). Standard procedures for consultations, interdisciplinarity, sufficient staff, and anamnesis with pro-active risk assessment were generally mentioned as CRM measures. Three sub-categories could be identified. The first was assaults by staff on patients during the therapeutic process (A1.2.1, e.g. sexual contacts or abuse of power by the therapist). Suggested as possible measures were, special training, intervision (peer consulting) and supervision for staff, the recommendation to avoid one-to-one consultations, and the implementation of an ombudsman service that a patient can turn to.

The second sub-category was diagnostic errors (A1.2.2). This encompasses the misdiagnosis of a mental illness when it was a physical illness and the misdiagnosis of psychiatric illnesses [cf. [13]. This can result in incorrect treatment (therapy, medication) that can worsen the patient’s condition. Differential diagnoses are crucial to prevent diagnostic errors. Thus, many psychiatric hospitals use specific instruments to differentiate between physical and mental diagnoses.

The third sub-category concerns specific medication risks occurring mainly in psychiatry (A1.2.3). Here, side-effects of medication are most important (e.g. weight gain, loss of libido), as they are a primary reason for patients being non-compliant and not taking their medications. Another risk is apparent if patients accumulate medications for substance abuse or with the intention to commit suicide. Therefore, patients should be informed and educated about medications and their possible effects and side-effects, and patients’ needs should be clarified and taken into account. The distribution and intake of medication needs to be monitored rigorously.

The interviews showed that this very mental-health specific topic of errors in the process of therapy, especially in psychotherapy, is insufficiently discussed and still rather vague. Treatment errors are seldom recognized or if they are, it is often too late, as therapy deals with the psyche and not with the observable body. In mental health care it can even be that a patient is judged to be “resistant to therapy, something that would never be accepted for a knee injury” (I3, P70). Furthermore, there are often different ideas among the mental health care professionals of what the right therapy might be for which illnesses. In addition, sometimes it is “rather the environment and not the patient that needs treatment” (I6, P53).

#### A1.3) Risks associated with mental illnesses

The third focus regarding specific clinical risks in mental health care was on risks associated with mental illnesses (A1.3, n=37). This contains mentions of particular illnesses (e.g. addiction, acute psychosis, mania, depression, anxiety disorders, or personality disorders) that might increase the possibility for certain risks (e.g. violent behavior or suicide). Risks associated with schizophrenic/psychotic disorders were mentioned most frequently. Most private psychiatric hospitals in our sample select patients according to their mental illnesses as they are not obligated to accept all patients (in contrast to public hospitals). For example, patients with psychoses, addiction or major depression may not be accepted by a private hospital; thereby minimizing possible risks for the hospital. Overall, tools to assess the level of depression, suicidal tendencies, violence, etc. are most important to identify risks.

Most interviewees also mentioned that many psychiatric patients (“15-18%”, I11, P117) are in hospital against their will (A1.3.1). The patients might have an involuntary commitment or do not believe that they are ill, which can result in violence, compulsory measures (see above) or leaving hospital against medical advice. Another risk is substance abuse and its consequences (A1.3.2) if, for example, drugs and injection devices (e.g. syringes) are smuggled into the hospital. CRM practices mentioned are to require patients to sign a binding addiction contract and to search patients to prevent them from smuggling drugs into the hospital.

#### A1.4) Leaving hospital against medical advice (Absconding)

Six out of 11 interviewees mentioned leaving hospital against medical advice or absconding from the hospital as another specific risk in mental health care (A1.4, n=9). There are various reasons why a patient might want to escape from a psychiatric hospital. It can be a consequence of the mental illness (e.g. hearing imperative/bidding voices that command a patient to escape) or because a patient is hospitalized against his/her will (see above). An escape from treatment might have severe consequences (e.g. (attempted) suicide, assault). CRM measures mentioned were the internal transfer of endangered patients to a closed ward, a very close observation/support of the patient and, if the patient did escape, a search by police.

### A2) Clinical risks in common with medical health care

All interviewees also mentioned clinical risks that are known in medical health care but are also important in mental health care (A2, n=106). They are described briefly as they are well documented in the literature and not the focus of this study. Medication risks were mentioned most frequently (A2.1, n=33): confusion of medication, incorrect dose, incorrect administration, etc. Some interviewees judged medication risks to be just as important as in medical health care, whereas others found them not to be as critical in mental health. Infections and hygiene (A2.2, n=26) were also mentioned, but were not considered as important as in medical health care. One reason for this being that psychiatric hospitals have no surgery. Falls (A2.3, n=12) were also a topic in some interviews, especially regarding geronto-psychiatry or in the context of withdrawal symptoms.

Risky organizational and technological conditions that influence patient safety were also mentioned. Staff risks (A2.4) were identified, including staff shortage, too many shift changes, and stress and workload often resulting in prolonged absences from work and high staff turnover. Some interviewees saw this as a problem specific to mental health care as staff absenteeism due to illness was judged as being much more common than in other domains, including medical health care. Regarding technology and equipment (A2.5), correct application and periodic maintenance were seen as being most important. A high rate of internal patient transfers (A2.6) was also seen as potentially risky as primary caregivers change, knowledge about the patient is lost and handovers must be organized.

### B) Other risks (non-clinical)

All interviewees also mentioned non-clinical risks that are mostly not specific for mental health care (B, n=47). Economic, construction, infrastructural and fire risks were mentioned. These risks were not classified further because this was not the focus of this study. However, some risks, such as data protection (to protect patients from stigmatization), or risks relating to hospital image (to avoid negative press) were judged to be especially important for psychiatric hospitals.

### C) Risks for the staff

Staff safety is an important topic in psychiatric hospitals and all interviewees explicitly referred to it (C, n=38). It is specific to mental health care insofar as staff face risks, such as aggression and violence, far more often than in medical health care. A prospective 1998 study in six psychiatric hospitals captured all obvious aggressive physical contacts over six months: 144 assaults on 170 members of staff were found [28]. “Working for 8 or more hours a day and being constantly conscious of the possibility of violence, I think, is almost unacceptable” (I2, P94). This can lead to work stress, burn-out and prolonged absenteeism from work due to illness (see above). “We have more than 25% drop-outs because of staff illnesses; this is a very high number” (I10, P55). Fellow staff members and patients suffer from such situations. Staff can also become a second victim [29] as (attempted) suicides, diagnostic errors, medication errors or performing compulsory measures can be enormously burdensome. Therefore, staff and patient safety are closely interrelated and affect each other, at least partially.

## Discussion

This study offers, for the first time, an overview of the main risk themes of CRM in mental health care and is independent of specific hospitals. The overview augments previous research, as it is systematic, exhaustive, and does not focus on selected risks. The result of counting the risks indicates which risks are common and important. Whereas medication errors are in the uppermost position of risks to patients in hospitals for physical disorder [cf. [1,8], CRM in mental health is first concerned with violence and self-harm. Self-destructive behavior (mainly suicide and attempted suicide) was mentioned the most, followed by violence/aggression from patients against others. In terms of CRM, this implies that the main goal, above all, is to protect patients and staff from other patients, as well as to protect patients from themselves [cf. [15]. Professional interventions can reduce violence in many cases. Important to achieving this are sensitization, education and training of staff as well as the use of preventive instruments to predict violence. If something is happening, de-escalation (to calm the patient), diversion, and engagement are recommended as proactive interventions [12]. The consideration between the surveillance of the patient and the possibility to allow the patient to move freely remains a particular problem. Permanent surveillance increases safety and prevents suicides, but the patient is literally imprisoned and the necessary staff resources for the hospital to achieve this are enormous [30]. Therefore, striking the right balance between safety and freedom is also one of the delicate challenges in mental health care.

The second main risk theme concerns treatment errors. In particular, errors in the process of therapy, notably in psychotherapy, are insufficiently discussed and still rather vague (see results above, A1.2) so need further investigation. Diagnostic errors were seldom mentioned and seem to be neglected and underestimated similarly as is the case in medical health care. Despite the fact that they account for about 15% of medical errors and are the leading cause of medical malpractice litigation (twice as many cases as medication errors), diagnostic errors receive little attention [cf. [31,32]. This is probably because they are hard to measure, there being little data of incidence available, and because it is sometimes difficult even for experts to agree on the right diagnosis. However, especially in mental health care, where an incorrect diagnosis can result in incorrect therapy and prolonged stays in the hospital (sometimes for years), sensitization of staff and taking diagnostic errors into account in CRM is essential.

The third specific risk theme was risks associated with mental illnesses, such as psychosis or depression. Furthermore, many psychiatric patients lack insight regarding their illness and do not themselves think that they are ill and are hospitalized against their will. Therefore, due to their illnesses, most patients in mental health care differ greatly from patients in medical health care. Staff safety is directly related to the specifics of mental illnesses and is, as shown, a central theme in mental health care. These are additional main reasons why CRM in mental health care needs specialized concepts and strategies that complement the knowledge from CRM in medical health care. Some clinical risks such as medication risks, infections, hygiene, and falls, are common to various specializations in health care, and would benefit from the application of similar CRM practices.

### Limitations

A qualitative approach allows for the exploration of a subject where there is limited previous research. Although this approach proved to be valuable, the data were constrained by the number of participants available for interview. Therefore, the results may not be fully generalizable to all types of mental health hospitals (e.g. psychiatric units for geriatric or pediatric patients) and to other types of hospitals. Secondly, it is possible that the interviewees did not verbalize the full extent of their knowledge because of memory limitations and the fact that not all knowledge is conscious. These limitations are common in many qualitative studies [cf. [13]. However, the expert status and the diversity of the chosen interviewees guaranteed a thorough and expansive view of the subject.

Remarkably, interviewees only mentioned risks in inpatient psychiatry restrained to the period between admission and discharge of patients. The handovers from ambulatory to in-patient as well as the after-care were not discussed. For example, how does one ensure that a patient does not relapse promptly upon discharge only to be readmitted to the hospital? This situation mainly occurs if the ambulatory care setting is not clear, if a patient returns to his or her usual environment or if medications are discontinued.

## Conclusions

The current study adds to the understanding of patient safety and raises awareness for clinical risks in mental health. It uses expert interviews as an empirically sound way of generating knowledge in an emerging field that suffers from a shortage of research activity and empirical evidence. The overview of the main risk themes of CRM in mental health care and the proposed organizational CRM practices offer a valuable basis for CRM in psychiatry and an addition to CRM in hospitals in general. Psychiatric hospitals can use the overview to review the completeness of their assessment and knowledge of risks. It can also be used to prioritize the risks that need to be addressed. The CRM practices mentioned in the interviews provide guidance on how to deal with these risks. These guidelines may also be supplemented with a further step, for example by using a quantitative survey to gather information on the probability of occurrence and severity of individual risks, and to collect information about the most effective and most feasible measures. Overall, research and knowledge of patient safety is growing. CRM offers an essential contribution as it aims to reduce harm to patients [8]. Studying CRM in particular settings, such as mental health care, is imperative in order to build safer health systems and to improve safety in general, but also for patients in mental health, whose illnesses render them extremely vulnerable.

## Abbreviations

CRM: Clinical risk management; I1, P3: This index number indicates the interview and the paragraph where a quote was found.

## Competing interests

The authors declare that they have no competing interests.

## Authors’ contributions

MB and TM designed and executed the project. MB drafted the initial manuscript. TM revised the manuscript critically for important intellectual content. All authors read and approved the final manuscript.

## Pre-publication history

The pre-publication history for this paper can be accessed here:

http://www.biomedcentral.com/1472-6963/13/44/prepub
